# Supplementary material for: The importance of biofilm formation for cultivation of a Micrarchaeon and its interactions with its Thermoplasmatales host
Source: Nat Commun. 2022 Apr 1;13:1735. doi: 10.1038/s41467-022-29263-y (PMC8975820; doi:10.1038/s41467-022-29263-y)
Supplement: Supplementary file 1 — Supplementary information [file 41467_2022_29263_MOESM1_ESM.docx]

**Supplementary information**

The importance of biofilm formation for cultivation of a Micrarchaeon and its interactions with its *Thermoplasmatales* host

Susanne Krause, Sabrina Gfrerer, Andriko von Kügelgen, Carsten Reuse, Nina Dombrowski, Laura Villanueva, Boyke Bunk, Cathrin Spröer, Thomas R. Neu, Ute Kuhlicke, Kerstin Schmidt-Hohagen, Karsten Hiller, Tanmay A. M. Bharat, Reinhard Rachel, Anja Spang and Johannes Gescher

**Suppl. Figure S1**


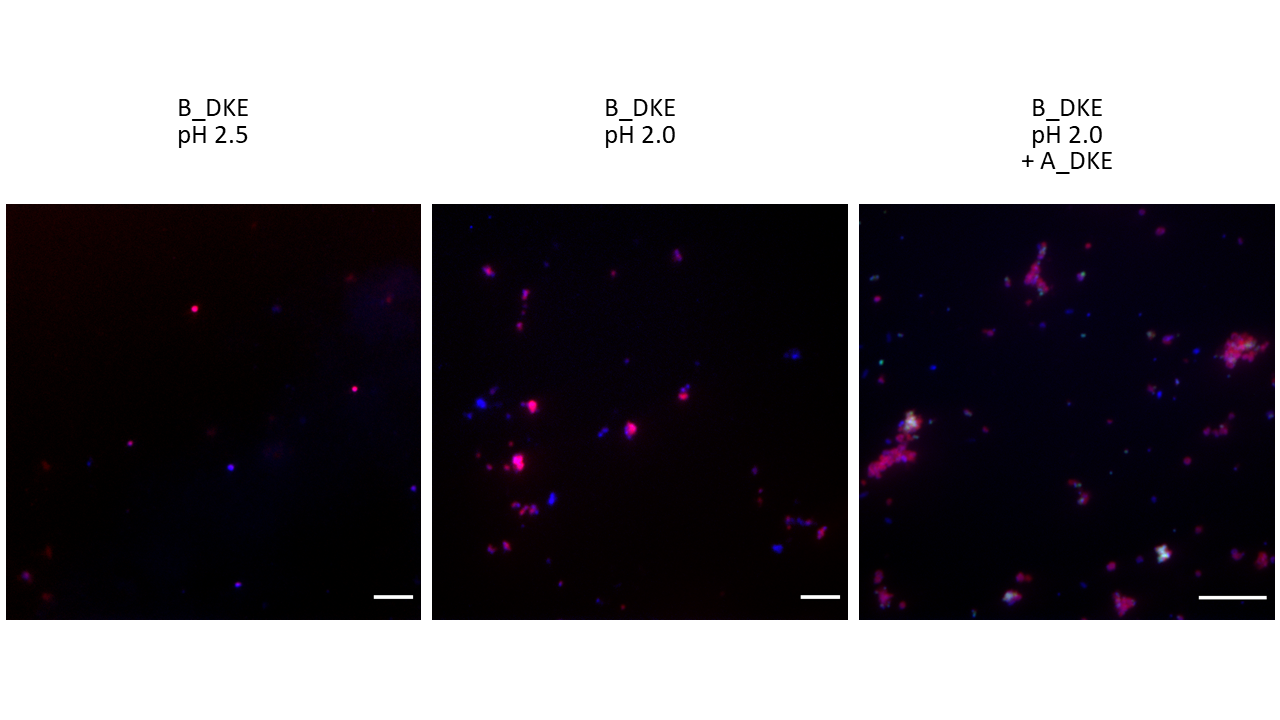


Suppl. Figure S1: CARD-FISH pictures depicting growth morphology of Ca. Scheffleriplasma hospitalis cells under different conditions. Microscopic pictures showing (from left to right) Ca. Scheffleriplasma hospitalis cultivated at pH 2.5, cultivated at pH 2.0 and co-cultivated with Ca. Micrarchaeum harzensis at pH 2.0. Samples were taken at similar growth phases. Shown are representative images of biological triplicates. Cells are stained as follows: Ca. Scheffleriplasma hospitalis in red (TH1178 probe), Ca. Micrarchaeum harzensis in green (ARM980 probe) and all cells in blue (DAPI). Scale bars equal 10 µm.

**Suppl. Figure S2**

Suppl. Figure S2: Growth curve of Ca. Scheffleriplasma hospitalis pure culture. Growth was estimated in amount of DNA in ng per mL culture (black squares) and Fe(II) concentration (light grey circles). Isolated DNA from growth curve samples was quantified via Qubit® ds DNA HS Assay kit and normalized to the culture volume used for DNA isolation. Data shown are mean values of a triplicate, error bars indicate standard deviations. Source data are provided as a Source Data file.

**Suppl. Figure S3**





Suppl. Figure S3: Depiction of the metabolic pathways of EPS precursors from fructose-6-phophate. The TPM values of the responsible enzymes are listed in green (Ca. Micrarchaeum harzensis), red (Ca. Scheffleriplasma hospitalis in co-culture) and orange (Ca. Scheffleriplasma hospitalis in pure culture). Abbreviations are; UTP: uridine 5’-triphosphate; UDP: uridine 5’-diphosphate; GlcNAc: N-acetyl-glucosamine; GalNAc: N-acetyl-galactosamine.

**Suppl. Figure S4**

Suppl. Figure S4: Fe(II) concentration during growth curve of co-culture of Ca. Micrarchaeum harzensis and Ca. Scheffleriplasma hospitalis and pure culture of Ca. Scheffleriplasma hospitalis for metabolic analysis. Data shown, are mean values of a triplicate, respectively. Error bars indicate standard deviations. Black boxes indicate time points used for analysis shown in figure 6, grey colouration indicates Fe^2+^ concentration of samples used for transcriptomic analysis. Source data are provided as a Source Data file.

**Suppl. Figure S5**


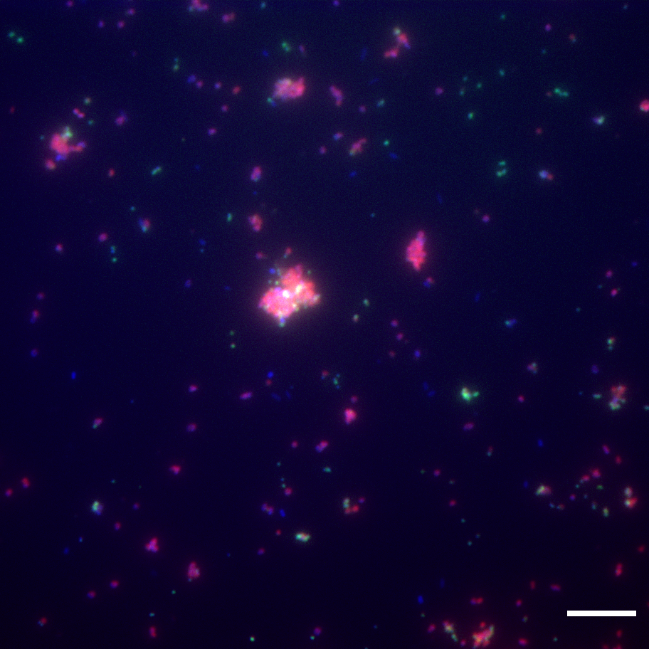

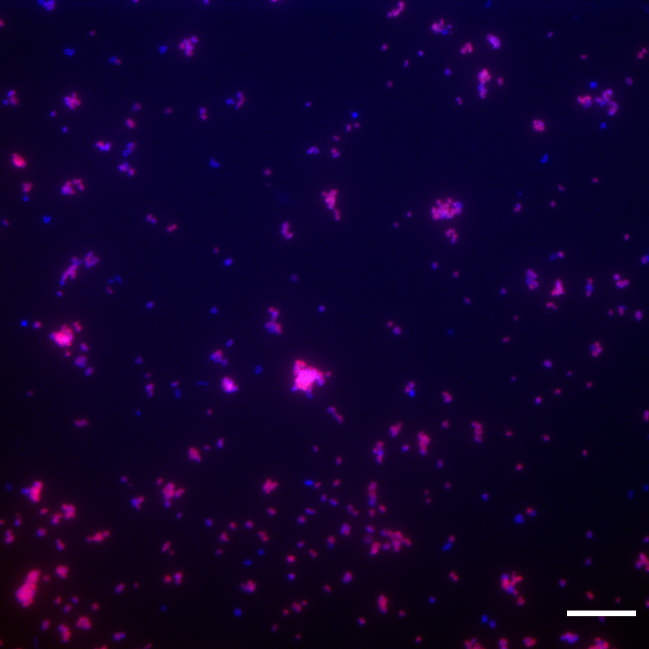


Suppl. Figure S*5*: CARD-FISH pictures of cultures used for lipid analysis. Left picture shows an overlay of the used co-culture containing *Ca*. Micrarchaeum harzensis and *Ca.* Scheffleriplasma hospitalis, right pictures an overlay of the pure culture of *Ca.* Scheffleriplasma hospitalis. Red: TH1178 probe staining *Ca*. Scheffleriplasma hospitalis, green: ARM980 probe staining *Ca.* Micrarchaeum harzensis, blue: DAPI as counter staining. Scale bars equal 10 µm. Shown are representative images of 40 micrographs taken, respectively.
