# Supplementary material for: The importance of biofilm formation for cultivation of a Micrarchaeon and its interactions with its Thermoplasmatales host
Source: Nat Commun. 2022 Apr 1;13:1735. doi: 10.1038/s41467-022-29263-y (PMC8975820; doi:10.1038/s41467-022-29263-y)
Supplement: Supplementary file 2 — Reporting Summary [file 41467_2022_29263_MOESM2_ESM.pdf]

nature portfolio

Corresponding author(s): Johannes Gescher

Last updated by author(s): Johannes Gescher

Reporting Summary

Nature Portfolio wishes to improve the reproducibility of the work that we publish. This form provides structure for consistency and transparency in reporting. For further information on Nature Portfolio policies, see our Editorial Policies and the Editorial Policy Checklist.

Statistics

For all statistical analyses, confirm that the following items are present in the figure legend, table legend, main text, or Methods section.

n/a	Confirmed	
☐	☒	The exact sample size (*n*) for each experimental group/condition, given as a discrete number and unit of measurement
☐	☒	A statement on whether measurements were taken from distinct samples or whether the same sample was measured repeatedly
☐	☒	The statistical test(s) used AND whether they are one- or two-sided<br>*Only common tests should be described solely by name; describe more complex techniques in the Methods section.*
☐	☒	A description of all covariates tested
☐	☒	A description of any assumptions or corrections, such as tests of normality and adjustment for multiple comparisons
☐	☒	A full description of the statistical parameters including central tendency (e.g. means) or other basic estimates (e.g. regression coefficient) AND variation (e.g. standard deviation) or associated estimates of uncertainty (e.g. confidence intervals)
☐	☒	For null hypothesis testing, the test statistic (e.g. *F*, *t*, *r*) with confidence intervals, effect sizes, degrees of freedom and *P* value noted<br>*Give P values as exact values whenever suitable.*
☒	☐	For Bayesian analysis, information on the choice of priors and Markov chain Monte Carlo settings
☐	☒	For hierarchical and complex designs, identification of the appropriate level for tests and full reporting of outcomes
☒	☐	Estimates of effect sizes (e.g. Cohen's *d*, Pearson's *r*), indicating how they were calculated

Our web collection on statistics for biologists contains articles on many of the points above.

Software and code

Policy information about availability of computer code

Data collection	Leica LAS AF 6000, SMRT Portal version 2.3.0, Illumina NextSeq® 500 NGS, Agilent 1260 UHPLC, SerialEM v. 3.8, Agilent 7890A-Agilent 5975C (GC-MS) + Gerstel MPS2 (autosampler), Axio Vision 4.7
Data analysis	Burrows-Wheeler Aligner bwa 0.6.2, Prokka 1.8, Hmmsearch v3.1b298, custom scripts available at https://zenodo.org/record/3839790, BLASTp v2.7.1, InterProScan v5.29-68.0, MAFFT L-INS-i v7.407, BMGE v1.12, IQ-TREE v1.6.10, MAFFT v7.407, TrimAL v1.2rev59, FastTree v2.1.10, Kallist v0.45.0, CLC Genomics Workbench 20.0.1 and Create Expression Browser 1.1 implementation, MetaboliteDetector software, IMOD 4.12.10 - 4.12.16/4.10.43, CTFFIND4, Tomo3D 2.0, Fiji 2.3.0/1.53f, R-studio

For manuscripts utilizing custom algorithms or software that are central to the research but not yet described in published literature, software must be made available to editors and reviewers. We strongly encourage code deposition in a community repository (e.g. GitHub). See the Nature Portfolio guidelines for submitting code & software for further information.

Data

Policy information about availability of data

All manuscripts must include a data availability statement. This statement should provide the following information, where applicable:

- Accession codes, unique identifiers, or web links for publicly available datasets
- A description of any restrictions on data availability
- For clinical datasets or third party data, please ensure that the statement adheres to our policy

The genome sequences generated in this study including annotations have been deposited at NCBI Genbank under Accession Numbers CP060530 [https://www.ncbi.nlm.nih.gov/nuccore/CP060530.1] and CP060531 [https://www.ncbi.nlm.nih.gov/nuccore/CP060531.1]. Raw reads of transcriptomic data generated in this study are available asunder SRA files under BioSample accession codes SAMN15702898 [https://www.ncbi.nlm.nih.gov/biosample/15702898] and

SAMN15702859 [https://www.ncbi.nlm.nih.gov/biosample/15702859]SRX8933312-SRX8933318. All generated raw files of phylogenetic trees and generated MS raw data of the lipid analysis can be found in a repository (doi: 10.5281/zenodo.4725436) [https://zenodo.org/record/4725436]. The MS raw data for metabolome analysis can be found in a repository [https://doi.org/10.24355/dbbs.084-202202151452-0]. Reference genomes and primers used in this study are provided in the Supplementary Information. Source data are provided with this paper.

Field-specific reporting

Please select the one below that is the best fit for your research. If you are not sure, read the appropriate sections before making your selection.

- [x] Life sciences
- [ ] Behavioural & social sciences
- [ ] Ecological, evolutionary & environmental sciences

For a reference copy of the document with all sections, see nature.com/documents/nr-reporting-summary-flat.pdf

Life sciences study design

All studies must disclose on these points even when the disclosure is negative.

Sample size	Our study is based on data from at least independent triplicates, which is standard in microbiological studies. Although I cannot provide a specific reference for this, I would also like to highlight that the standard of our manuscript regarding replicates is higher compared to other studies conducted with Micrarchaeota. A recent study by Sakai et al. (https://dx.doi.org/10.1073%2Fpnas.2115449119) published in PNAS was for instance not using any kind of replicate data.
Data exclusions	No data was excluded from the study.
Replication	The data is based on reproducible replicate (at least triplicate) data.
Randomization	Randomization is not relevant for this manuscript as we were characterizing and comparing only two different cultures in at least triplicate experiments.
Blinding	In our research we studied and compared the characteristics of a microbial pure culture and a co-culture. Both cultures had to be continously screened for the quantity of the individual microbes in order to take samples for further analysis. Hence, blinding would not have been useful here. The same is true for all other experiments in which we always assessed the quantity of the microorganisms as one parameter. Hence, blinding would simply not been possible here.

Reporting for specific materials, systems and methods

We require information from authors about some types of materials, experimental systems and methods used in many studies. Here, indicate whether each material, system or method listed is relevant to your study. If you are not sure if a list item applies to your research, read the appropriate section before selecting a response.

Materials & experimental systems

n/a	Involved in the study	
[x]	[ ]	Antibodies
[x]	[ ]	Eukaryotic cell lines
[x]	[ ]	Palaeontology and archaeology
[x]	[ ]	Animals and other organisms
[x]	[ ]	Human research participants
[x]	[ ]	Clinical data
[x]	[ ]	Dual use research of concern

Methods

n/a	Involved in the study	
[x]	[ ]	ChIP-seq
[x]	[ ]	Flow cytometry
[x]	[ ]	MRI-based neuroimaging
