# Supplementary material for: The importance of biofilm formation for cultivation of a Micrarchaeon and its interactions with its Thermoplasmatales host
Source: Nat Commun. 2022 Apr 1;13:1735. doi: 10.1038/s41467-022-29263-y (PMC8975820; doi:10.1038/s41467-022-29263-y)
Supplement: Supplementary file 4 — Description of Additional Supplementary Files [file 41467_2022_29263_MOESM4_ESM.pdf]

Title: Supplementary Data 1
Description: Genes of *Ca.* Micrarchaeum harzensis with a function in central metabolic pathways with gene ID and annotation. Annotations according to Suppl. Data S5 and S6.

Title: Supplementary Data 2
Description: The table lists all used lectins with carbohydrate binding specificity, linkage type, fluorescence label as well as supplier. Lectins, which bound either *Ca.* Micrarchaeum harzensis and/or *Ca*. Scheffleriplasma hospitalis EPS are marked in light orange.

Title: Supplementary Data 3
Description: Genes in *Ca.* Micrarchaeum harzensis and *Ca*. Scheffleriplasma hospitalis genome with function in glycosylation and synthesis of carbohydrate precursors for EPS matrix. Listed are the gene ID, gene product, arCOG/TIGR numbers and e-values, respectively, as well as TPM expression values in co- and pure culture. Annotations were done with KEGG Annotation Server and compared to results of Suppl. Data S5-S6.

Title: Supplementary Data 4
Description: Relative abundance of lipids in co-culture (*S. hospitalis*+*M. harzensis*) and pure culture (*S. hospitalis*). Abbreviations are CL: core lipids, IPL: intact polar lipids.

Title: Supplementary Data 5
Description: Annotation of protein files of *Ca.* Micrarchaeum harzensis and Micrarchaeota reference genomes across different databases. The table was created as described in Methods section Genome annotations.

Title: Supplementary Data 6
Description: Annotation of protein files of *Ca.* Scheffleriplasma hospitalis and *Thermoplasmatales* reference genomes across different databases. The table was developed as described in Methods section Genome annotations.

Title: Supplementary Data 7
Description: Genes used for phylogenetic analyses in this study.

Title: Supplementary Data 8
Description: Reference genomes used for phylogenetic analyses in this study.

Title: Supplementary Data 9
Description: Primers used for calculation of the cell numbers of *Ca.* Micrarchaeum harzensis to *Ca*. Scheffleriplasma hospitalis via qPCR and monitoring of composition of cultures via PCR.

Title: Supplementary Movie 1
Description: Tomogram displaying a *Ca*. Micrarchaeum harzensis cell.

Title: Supplementary Movie 2
Description: Tomogram displaying a *Ca*. Scheffleriplasma hospitalis cell.

Title: Supplementary Movie 3
Description: Tomogram of Figure 8c displaying a *Ca*. Scheffleriplasma hospitalis cell interacting with two *Ca.* Micrarchaeum harzensis cells.
